# Epigenetic mechanisms of particulate matter exposure: air pollution and hazards on human health

**DOI:** 10.3389/fgene.2023.1306600

**Published:** 2024-01-17

**Authors:** Dulcemaría Gavito-Covarrubias, Ivonne Ramírez-Díaz, Josué Guzmán-Linares, Ilhuicamina Daniel Limón, Dulce María Manuel-Sánchez, Alejandro Molina-Herrera, Miguel Ángel Coral-García, Estela Anastasio, Arely Anaya-Hernández, Primavera López-Salazar, Gabriel Juárez-Díaz, Javier Martínez-Juárez, Julián Torres-Jácome, Alondra Albarado-Ibáñez, Ygnacio Martínez-Laguna, Carolina Morán, Karla Rubio

**Affiliations:** ^1^ International Laboratory EPIGEN, Consejo de Ciencia y Tecnología del Estado de Puebla (CONCYTEP), Instituto de Ciencias, Benemérita Universidad Autónoma de Puebla (BUAP), Puebla, Puebla, Mexico; ^2^ Universidad Popular Autónoma del Estado de Puebla (UPAEP), Puebla, Mexico; ^3^ Laboratory of Neuropharmacology, Faculty of Chemical Sciences, Benemérita Universidad Autónoma de Puebla (BUAP), Puebla, Mexico; ^4^ Centro de Investigación en Genética y Ambiente, Universidad Autónoma de Tlaxcala, Tlaxcala, Mexico; ^5^ Centro de Investigaciones en Dispositivos Semiconductores (CIDS), Instituto de Ciencias, Benemérita Universidad Autónoma de Puebla (BUAP), Puebla, Mexico; ^6^ Laboratorio de Fisiopatología Cardiovascular, Instituto de Ciencias, Benemérita Universidad Autónoma de Puebla (BUAP), Puebla, Mexico; ^7^ Vicerrectoría de Investigación y Estudios de Posgrado, Benemérita Universidad Autónoma de Puebla (BUAP), Puebla, Mexico; ^8^ Centro de Investigación en Fisicoquímica de Materiales, Instituto de Ciencias, Benemérita Universidad Autónoma de Puebla (BUAP), Puebla, Mexico

**Keywords:** air pollution, epigenetic regulation, cancer, neurotoxicity, metabolism, fibrosis, oxidative stress, transgenerational epigenetics

## Abstract

Environmental pollution nowadays has not only a direct correlation with human health changes but a direct social impact. Epidemiological studies have evidenced the increased damage to human health on a daily basis because of damage to the ecological niche. Rapid urban growth and industrialized societies importantly compromise air quality, which can be assessed by a notable accumulation of air pollutants in both the gas and the particle phases. Of them, particulate matter (PM) represents a highly complex mixture of organic and inorganic compounds of the most variable size, composition, and origin. PM being one of the most complex environmental pollutants, its accumulation also varies in a temporal and spatial manner, which challenges current analytical techniques used to investigate PM interactions. Nevertheless, the characterization of the chemical composition of PM is a reliable indicator of the composition of the atmosphere, the quality of breathed air in urbanized societies, industrial zones and consequently gives support for pertinent measures to avoid serious health damage. Epigenomic damage is one of the most promising biological mechanisms of air pollution-derived carcinogenesis. Therefore, this review aims to highlight the implication of PM exposure in diverse molecular mechanisms driving human diseases by altered epigenetic regulation. The presented findings in the context of pan-organic cancer, fibrosis, neurodegeneration and metabolic diseases may provide valuable insights into the toxicity effects of PM components at the epigenomic level and may serve as biomarkers of early detection for novel targeted therapies.

## 1 Introduction

One of the main pollutants suspended in the atmosphere is particulate matter (PM), whose sources are natural and anthropogenic. Physical and chemical characteristics of the PM subtypes vary considerably (Table 1), also depending on whether they undergo transformation into the atmosphere, thus being primary (without transformation) or secondary (they form in the atmosphere) (Mendoza et al., 2010). While size remains the central aspect of concern due to its harmful effect on the organism’s wellbeing, given its role in predisposing their chemical composition and behavior within the atmosphere and the environment, it is also necessary to consider PM density, humidity, and region-associated air currents.

**TABLE 1:** General properties of Particular Matter subtypes.

Source	Size diameter (µm)	Surface area per particle_1_	Particle number_2_	Fundamental composition	Characteristics	Examples
Natural Volcanic emissions, soil aerosol, dust storms, forest fires, pollen, and mold volcanic emissions	PM_1_	0.0001	1,000,000	Elemental carbon	- Contains more harmful substances on its surface	Pollen, fungal spores, bacteria
					- Could potentially enter the bloodstream	
	PM_2.5_	0.625	64	Sulfates	- Reaches the outer parts of the air passages	Volcanic ashes, soil particles, spores
					- Unable to get into the bloodstream	
	PM_10_	1	1	SiO_2_, Al_2_O_3_, Na_2_O, and CaO	- Trapped in the initial part of the air passages	Sandstorms, salt crystals
					- Might cause skin and mucous membrane irritation	
Anthropogenic Agricultural burning or waste incineration, biomass burning, wood burning, bark or road dust, cigarette smoke, cooking, construction, burning fossil fuels, home heating, industry, mechanical wear, power plants, sea salt, and transportation	PM_1_	0.0001	1,000,000	Elemental carbon	- Contains more harmful substances on its surface	Vehicle exhaust, industrial combustion, chemical reactions
					- Could potentially enter the bloodstream	
	PM_2.5_	0.625	64	Heavy metals, organic compounds, sulfates, nitrates, and ammonium	- Reaches the outer parts of the air passages	Wood stoves, heating methods, combustion of fossil fuels, waste incineration
					- Unable to get into the bloodstream	
	PM_10_	1	1	Pb, Cd, Zn, Cu, Ni, V, Sb	- Trapped in the initial part of the air passages	Mining and quarrying, landfills
					- Might cause skin and mucous membrane irritation	

^1^ Assuming uniform density and same mass distribution.

^2^ In an equal amount of mass.

## 2 General characteristics of particulate matter subtypes

### 2.1 Chemical and physical composition

Depending on their size, PM is classified as PM_10_, whose diameter is less than or equal to 10 microns, PM_2.5_ those with a diameter less than 2.5 microns, and PM_1_ to label those with a diameter less than 1 micron. The diameter of the PM predisposes the composition of the particles, thus PM_1_, known as ultrafine, displays a fundamental carbon composition, while fine PM_2.5_ generally contains heavy metals, organic compounds, sulfates, nitrates and ammonium. PM_1_ contains elements of fugitive construction dust as well as chemical elements disposed of by industries in areas of vehicular flow. In contrast, PM_10_ particles are predominantly composed of secondary particles (Kim et al., 2021). The characterization of the chemical composition of PM is a reliable indicator of the composition of the atmosphere, the quality of breathed air in urbanized societies, industrial zones and consequently gives support for pertinent measures to avoid serious health damage. In this sense, PM has been previously associated with the prevalence of genotoxic, mutagenic, or carcinogenic activities (Basith et al., 2022). Its composition varies because it depends on the different chemical combinations that occur along with variable factors including regions, climate, and anthropogenic activities. The natural PM sources include volcanic emissions, soil aerosol, dust storms, forest fires, pollen, and mold volcanic emissions. In contrast, anthropogenic origins are related to agricultural burning or waste incineration, biomass burning, wood burning, bark or road dust, cigarette smoke, cooking, construction, burning fossil fuels, home heating, industry, mechanical wear, power plants, sea salt, and transportation (Table 1) (Basith et al., 2022).

### 2.2 Molecular interactions

The characterization of the molecular interactions involving PM is a complex process. It comprises a variety of factors, such as the composition of the particles, size, humidity, air temperature, and the presence of other pollutants. The molecular interactions between PM and the human body are not fully understood. However, it is believed that PM can damage cells in the lungs and the respiratory tract, among other tissues, but also trigger inflammation therefore leading to a variety of health problems. For instance, PM can produce reactive oxygen species (ROS), which can lead to oxidative stress, inflammatory cell activation, and the release of proinflammatory cytokines. It can also trigger the recruitment of inflammatory cells to the airway through the activation of signaling pathways such Nrf2-Keap1-ARE, MAPK, and PI3K/Akt pathways (Liu et al., 2022a; Kim et al., 2022), as discussed in detail in the next sections.

**FIGURE 1:**
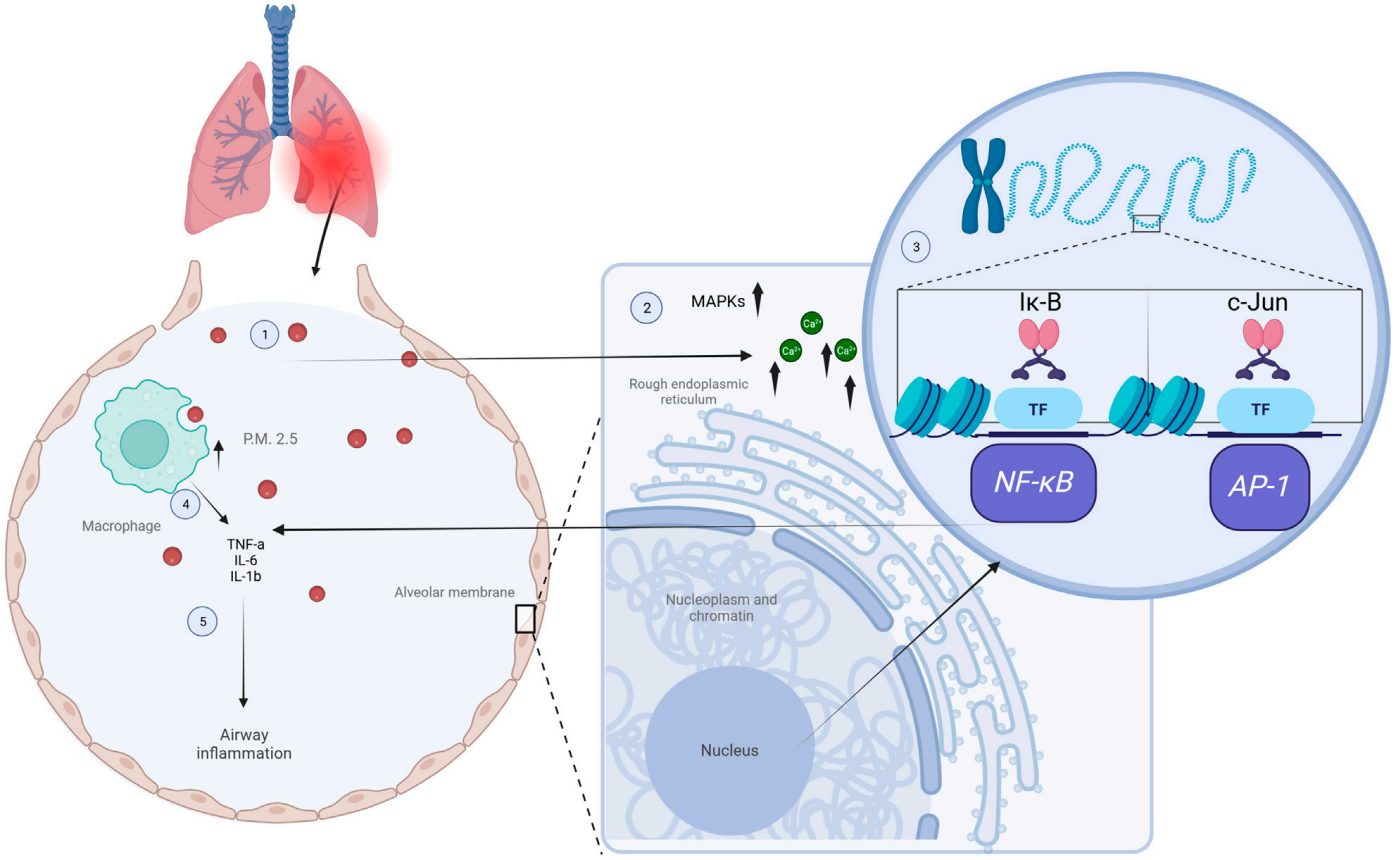
Impact of PM_2.5_ exposure on inflammatory processes. Inside the alveoli, PM_2.5_ particles are taken up by macrophages and alveolar cells through phagocytosis (1), leading to higher calcium release from the endoplasmic reticulum signaling pathways and causing phosphorylation of the MAPK family (2), which leads to enhanced gene transcription of *NF-κB* and *AP-1* by phosphorylation and degradation of *Iκ-B*, and phosphorylation and activation of c-Jun, respectively (3). This transcriptional activation promotes the release of proinflammatory cytokines like IL-6, IL-8, and TNF-α (4), which triggers a T2 inflammatory response and airway hyperreactivity (5). Figures created using Biorender, adapted from Liu et al. (2022a), licensed CC-BY 4.0, and Lee et al. (2023), licensed CC-BY-NC 4.0.

Recent studies have demonstrated the effectiveness of optical diffraction tomography (ODT) for real-time visualization and quantitative analysis of the molecular interactions between PM_2.5_ and individual living cells. For instance, PM_2.5_ exposure in airways resulted in increased levels of calcium release occurring from the endoplasmic reticulum and played a crucial role in the phosphorylation-dependent activation of the MAPK family, which leads to enhanced gene transcription of *NF-κB* and *AP-1*. This transcriptional activation in turn promotes the release of proinflammatory cytokines like IL-6, IL-8, and TNF-α, which trigger a T2 inflammatory response and induce airway hyperreactivity, as shown in Figure 1 (Liu et al., 2022a). Other authors observed that PM_2.5_ particles were predominantly internalized by macrophages through phagocytic behavior. Since macrophages play a crucial role in recognizing and processing PM_2.5_, these events reduced the risks associated with PM_2.5_ exposure. Moreover, the active interaction between macrophages and PM_2.5_ was evident as they underwent shape changes or migratory profiles to enhance PM_2.5_ uptake over time, suggesting that PM_2.5_ exposure might induce macrophage damage (Lee et al., 2023). In addition, this study monitored epithelial cells in real-time at 10-min intervals following PM_2.5_ treatment, highlighting the protective role of epithelial cells acting as a physical barrier and further protecting against the translocation of PM_2.5_ to other organs by internalization mechanisms such as phagocytosis (Lee et al., 2023).

Carbon nanoparticles and other components have diverse effects on lipid rafts, cellular membranes, and cellular processes through intricate interactions and molecular mechanisms. In airway epithelial cells, these nanoparticles modify lipid raft composition by elevating ceramide levels. Polycyclic aromatic hydrocarbons (PAHs), including benzo[a]pyrene (B[a]P), directly impact membrane fluidity and can interact with phospholipids, inducing changes in membrane structure and increased permeability (Peuschel et al., 2012; Rao et al., 2018). In a research focused on the role of PM_2.5_ during lung cancer progression, the authors found that PM_2.5_ exposure could activate the transcription factor Aryl hydrocarbon Receptor (AhR) in lung cancer cells, leading to the overexpression of *TMPRSS2* and the production of the pro-inflammatory cytokine IL-18. Then, activation of the AhR-TMPRSS2-IL-18 pathway promotes cell proliferation, invasion, and metastasis in lung cancer models. These findings suggest that PM_2.5_ exposure is a risk factor for lung cancer progression and that the AhR-TMPRSS2-IL18 pathway is a potential target for the development of new therapies against this disease (Wang et al., 2023). Moreover, in an innovative *in vitro* model that simulates cell interactions occurring in skin during atopic dermatitis, it was shown that PM_2.5_ exposure led to an upregulation of proinflammatory cytokines, including IL-1α, IL-1β, IL-6, IL-4, IL-13, and TNF-α in a co-culture assay (Roh et al., 2022).

Epigenetic damage is one of the most promising biological mechanisms of air pollution-derived carcinogenesis. DNA methylation, a common epigenetic modification primarily taking place in cytosines, particularly at CpG islands, is a crucial regulator of gene expression. Previous research has demonstrated that both short-term and long-term exposure to ambient air pollution are linked to alterations in DNA methylation levels. Methylation occurring in DNA repair genes, such as *ERCC1, ERCC6, OGG1, MGMT*, and *HMLH1* impacts their expression consequently influencing the repair mechanisms activated upon genetic damage caused by air pollution (Xu et al., 2022a). In a similar research, differential levels of DNA methylation were evidenced in *AHRR*, *COL5A1*, *TNS1*, and *LINC00886,* with their corresponding differential expression levels (Huang et al., 2021). The authors also analyzed the expression of DNA methyltransferases (DNMTs) and ten–eleven translocation (TET) enzymes, observing that a single treatment with PM_2.5_ for 24 h did not affect the expression of DNMTs, but led to a significant decrease in TET expression. However, when low concentrations of PM_2.5_ were administered daily for 7 days, the expression of *TET1*, *TET2*, and *TET3* was increased, suggesting differential effects upon acute or chronic exposure and the necessity of further studies (Huang et al., 2021).

The field of environmental epigenetics research has experienced a revolution due to the discovery of various ncRNA species, transforming our understanding of physiology and disease development (Frye et al., 2021; Olmedo-Suárez et al., 2022; Rubio et al., 2023a). ncRNAs, including miRNAs, piRNAs, and lncRNAs, play roles in biological processes by interacting with transcription factors and key regulatory proteins (Mattick et al., 2023). In particular, miRNAs are a crucial target of epigenetic damage caused by air pollution. miRNAs are a type of ncRNA that are on average 22 amino acids long and play important roles in gene transcription. Their mature forms can interact with ncRNAs inside the nucleus, and with the 3′untranslated region (3′UTR) of mRNAs in the cytoplasm, inducing degradation of the mRNAs and thus transcriptional and post-transcriptional repression (Singh et al., 2018; Rubio et al., 2019; Slack and Chinnaiyan, 2019).

### 2.3 Worldwide levels and seasonal variations

The presence of PM in the atmosphere represents a potential danger to ecosystems, human life, and plays an important role in climate change. This global issue is widely acknowledged and raises concerns on a global scale. The World Health Organization (WHO) estimates that air pollution causes about 12.6 million premature deaths per year worldwide (World Health Organization, 2016). According to the 2010 Global Burden of Disease study, outdoor air pollution is considered one of the leading causes of death globally, ranking among the top 10 risk factors worldwide. In developing countries located in Asia, it is one of the top 5-6 risk factors. Specifically in East Asia, outdoor air pollution was the fourth leading cause of mortality, while in South Asia it ranked as the sixth highest cause of death (Li et al., 2017). In 2019, non-communicable diseases caused 74% of all deaths globally, a significant increase compared to 54% in 1990. Ambient air pollution was the single largest environmental risk factor, responsible for roughly 50% of all deaths from environmental risk factors. More than 50% of deaths from ambient air pollution exposure occur in China and South Asia, and about 20% of the total global air pollution-related deaths occur in high-income countries in Europe and North America (Pozzer et al., 2023). The extensive evidence of health risks, even at very low concentrations of ambient PM_2.5_, remarks the urgency of adequate air quality management in all countries, regardless of their level of air pollution exposure (Pozzer et al., 2023). An analysis of 148 research studies, that included data from over 100 million people, found that short-term exposure to air pollution was associated with an increased risk of death from all causes. The risk of death was highest for exposure to PM_2.5_, followed by NO_2_. and O_3_. The risk of death was also higher in children and older adults and in people with chronic diseases. The risk of death was also higher in people who live in areas with high levels of air pollution. An interesting finding is that the risk of death increased by 1% for every 10 μg per cubic meter increase in PM_2.5_ levels, 0.5% for every 10 μg per cubic meter increase in NO_2_ levels, and 0.2% for every 10 μg per cubic meter increase in O_3_ levels (Orellano et al., 2020).

In this regard, the WHO collected and published on-the-ground measurements of air quality, specifically focusing on annual mean concentrations of PM_2.5_ and PM_10_. Currently, their primary purpose is to gather air quality data that can be analyzed to generate reliable estimates of population exposure for ambient air pollution in disease burden studies. In a recent update, they presented compelling evidence regarding the detrimental effects of air pollution on human health, even at lower concentrations than previously acknowledged. These guidelines recommend new air quality levels aimed at safeguarding population health, and the reduction of key air pollutants will also contribute to mitigating climate change. Notably, the guidelines establish new annual mean guideline values for 2021 of PM_2.5_ (5 µg/m^3^), which are half the previous official indicated value, 15 µg/m^3^ for PM_10_, and 15 µg/m^3^ for NO_2_ (World Health Organization, 2022). Their database contains data on air quality from over 6,000 monitoring stations in 117 countries. Compiled data shows that air pollution levels are still too high in many parts of the world, and that they are having a significant impact on human health. In the annual PM_10_, PM_2.5_ and NO_2_ means and data accessibility in the period of 2010–2019 by region, in human settlements of all sizes, PM_10_ levels in the Eastern Mediterranean and South-East Asia regions were higher than the global average. These levels exceeded the Air Quality Guidelines (AQG) by six to eight times. It is worth noting that these regions receive significant amounts of desert dust particles, which contribute to the elevated PM_10_ levels. Similarly, the PM_2.5_ levels followed a similar pattern, with the African and Western Pacific regions experiencing levels nearly five times higher than the AQG (World Health Organization, 2022).

Globally, only the population of 10% of the assessed settlements was exposed to annual mean levels of PM_10_ or PM_2.5_ that complied with the AQG. The proportion increased to 31% for interim target 4 (i.e., IT-4: 20 μg/m^3^ for PM_10_ and 10 μg/m^3^ for PM_2.5_) of the AQG, 54% for IT3 (30 μg/m^3^ for PM_10_ and 15 μg/m^3^ for PM_2.5_), 70% for IT2 (50 μg/m^3^ for PM_10_ and 25 μg/m^3^ for PM_2.5_) and 81% for IT1 (70 μg/m^3^ for PM_10_ and 35 μg/m^3^ for PM_2.5_) (World Health Organization, 2022). In a review focused on environmental pollution and toxicology, India and other countries were pointed out as those displaying differential variations in PM levels in winter (higher pollution) compared to the rainy season (lower contamination). In countries like China, seasonal variations were distinct too, with maximum concentrations during the winter and lowest ones during the summer. A higher exceedance on PM_2.5_ concentrations was mostly in the northern part compared to the southeast and west, where exceedance rates were more pronounced during the winter compared to the summer season (Mukherjee and Agrawal, 2018). A similar scenario was reported in an article that provides insights into the seasonal variations of major air pollutants and the long-range transport of PM_10_ in an urban environment with specific climate conditions in Transylvania, Romania. It was reported that during warmer periods, particularly in the summer, the concentrations of pollutants are lower. On the other hand, during the winter heating season, the pollutants exhibit significantly higher concentrations. This is mainly attributed to seasonal variations in energy use, specifically biomass burning, and the stability of the atmosphere (Bodor et al., 2020). Biomass burning and atmospheric stability contribute to significantly higher concentrations of pollutants during the winter heating season. Taken together, these results suggest that higher pollution levels are typically seen during the winter season, while lower contamination occurs during the rainy season or summer.

## 3 PM accumulation in human tissues

### 3.1 Cardiovascular system

Since small particles of ≤10 µm are found in the air, it is believed that the only organ that can be damaged is the lung and therefore could only induce respiratory diseases. However, epidemiological studies have reported a relationship between PM_2.5_ and the cardiovascular system resulting in hypertension, atherosclerosis, myocardial infarction, heart attacks, and alterations in heart rate (Barr et al., 2012; Jacobs et al., 2012; Madrigano et al., 2013; Sivakumar et al., 2022; Mordukhovich et al., 2015; Kirrane et al., 2019; Hayes et al., 2020). Also, several studies reported the presence of electrocardiogram alterations such as ST-segment changes and T-wave amplitude alterations (Chuang et al., 2008; Gardner et al., 2014). Chen *et al.* emphasized that PM_2.5_ impacted heart rate variability in humans, a significant indicator of cardiac impairment and heightened risk of fatal ventricular arrhythmias (Chen et al., 2017a). These changes were also reported to be linked to elevated levels of D-dimer and C-reactive protein, to finally conclude that long-term exposure to PM produces thrombotic and inflammatory processes, and an imbalance between the heart and autonomic nervous system interactions, ultimately causing changes in heart rate variability.

Further research has employed the C57BL/6 murine mouse to characterize the PM_2.5_ impact in the cardiovascular system (Wold et al., 2012). In this work, it was reported that PM_2.5_ exposure resulted in increased hypertrophic markers, fibrosis, diastolic dysfunction and ventricular remodeling. Mechanistically, PM exposure is associated with micelles around cardiac and pulmonary cells in the bloodstream. Thus, exposure to air pollution causes a disturbance in the extracellular space that allows particles to interact with the physiological processes of different cell types. This interaction depends on the responsive receptor on the cell membranes and its physicochemical properties. PM_2.5_ reaches the cardiovascular system after being introduced through the respiratory system, as this material is inhaled and deposited in the pulmonary alveoli (Pan et al., 2023). PM_2.5_ enters human cells by mechanisms such as phagocytosis or receptor-mediated endocytosis (RME, Figure 1) (Long et al., 2005; Yamagishi et al., 2022; Zhao et al., 2022).

The inhalation of PM_2.5_ causes oxidative stress and systemic inflammation, activating immune receptors and inflammatory mediators. These processes release pro-inflammatory and pro-oxidative molecules into the bloodstream, causing cardiovascular disease. Additionally, ion channels like TRPA1 and TRPV1 can be activated. This inflammation leads to the release of adhesion molecules resulting in leukocyte and platelet binding and activation of systemic blood coagulation. This observation is consistent with previous research linking elevated PM_2.5_ levels to increased formation of thrombin and hypercoagulation markers (Basith et al., 2022).

### 3.2 Respiratory system

The cilia and mucous in the nasal mucosa help the nose filter out the dust and dirt that we breath in. However, smaller particles (2.5 µm and below) cannot be filtered and can pass through and access the lower airway (Mo, 2019). A model has been proposed by several researchers for PM_2.5_ deposition in the acinar region, in which the movement of particles is primarily influenced by two factors: gravitational sedimentation and Brownian motion. Larger particles (greater than 2.0 μm) are predominantly affected by gravitational sedimentation, where their motion is governed by gravity force pulling them downward. On the other hand, Brownian motion—which results in random and erratic movements as a result of collisions with gas molecules—strongly influences smaller particles (less than 0.1 μm).

However, in a previous study a fluorescent imaging-based method was developed to observe PM_2.5_ deposition in mouse lungs and they found a non-uniform deposition pattern with a rate significantly higher than predicted (Li et al., 2019). This implies that the specific dynamics of particle movement in this size range are determined by irreversible processes occurring within the air sacs of the lungs, such as airflow patterns, local turbulence, or interactions with the lung surfactant (Li et al., 2019). Alternatively, it was found in 2020 that submicron PM containing Cd, Cu, Pb, and Zn are likely to deposit in the alveolar region, while the coarse particles contribute to the deposition of heavy metals in the head airway region, including the nose, mouth, pharynx, and larynx (Guo et al., 2021).

Given the heterogeneity of compounds that conform the PM, they could impact various biological activities, leading to changes in cytokine production, balance and function of some coagulation factors, pulmonary function as seen in the reduced forced vital capacity in patients with pulmonary fibrosis, respiratory symptoms, and cardiac function. As they pass through the airways, they can cause alterations such as recruiting inflammatory cells, and triggering the release of cytokines and reactive oxygen species (ROS). These inflammatory mediators can activate different pathways, including MAP kinases, NF-κB, and STAT1, or even induce DNA adducts (Falcon-Rodriguez et al., 2016; Winterbottom et al., 2018; Kyung and Jeong, 2020). The activation of the inflammatory pathways leads to a process of epithelial-mesenchymal transition, causing a dysfunction of the epithelial barrier through a series of underlying molecular events that end up with the disruption of adherent junctions, such as the upregulation of N-cadherin and vimentin and the repression of E-cadherin, ZO-1, and claudin-1 (Xu et al., 2019a). This dysfunction has been linked to an augmented absorption and translocation of the PM deposited along the respiratory epithelium that could even reach the brain via the olfactory bulb and other extrapulmonary tissues (Garcia et al., 2015; Chew et al., 2020), with multi-organic consequences that have not yet been explored. In conclusion, it has been proposed that Akt inhibition and ROS scavenging may represent potential targets for preventing the activation of these pathways and maintaining normal permeability (Lee et al., 2020).

### 3.3 Gastrointestinal system

Exposure to fine particles like PM_2.5_ or PM_10_ contained in polluted air produces alterations in several mechanisms related to organ pathogenesis, and ultimately to disease (Pope et al., 2015). Either ingestion or inhalation pathways are the most direct routes for PM_2.5_ and PM_10_ uptake (Pope et al., 2015), including the gastrointestinal system as the first contact with the external environment. It also enables PM absorption in the stomach mucosa and across the endothelial tissue in the gastrointestinal system (Feng et al., 2020). The oral cavity represents a delivery pathway to circulation for PM_2.5_, which then targets other organs. However, specific mechanisms are not yet understood. Presumably, PM_2.5_ accumulates in the gut microbiome (Bäumler and Sperandio, 2016) to produce diseases such as inflammation, obesity, metabolic syndrome, diabetes mellitus, and carcinogenesis (Säynävälammi et al., 1986).

Research groups explored how PM affects the intestine via oral mucociliary transport (Liu et al., 2021a). They showed disordered intestinal and brain microbiota, damaged permeability of the gut epithelial barrier, inflammatory responses, and oxidative stress. Six principal phyla of microbiota were affected by PM_2.5_, including a decrease in *Bacteroidetes* and an increase in *Firmicutes*, mainly in the genus Enterobacteriaceae (Karl et al., 2018; Liu et al., 2021a). Also, short-term exposure to PM_2.5_ produced mucosal injury while long term exposition produced alterations in crypt and villus integrity in the proximal and middle intestine. Immune infiltration cells were present in both expositions, although in the short-term they displayed higher infiltration patterns (Hu et al., 2022; Ohlsson et al., 2022). Moreover, alterations in *Firmicutes*, *Bacteroidetes*, *Proteobacteria* and *Tenericutes* correlated with additional metabolic pathways (Bäumler and Sperandio, 2016; Bajinka et al., 2020). The major changes observed in the metabolic pathway involved ABC transporters, cysteine and L-homocysteine, and the neuroactive ligand-receptor interaction (Blachier et al., 2021; Liu et al., 2021b).

PM_2.5_ increased the production of bacterial metabolites, such as glutamate, which is used by enterocytes for protein synthesis and energy balance (Blachier et al., 2009; Liu et al., 2021b). Linoleic acid also increased due to PM_2.5_ and produced an imbalance in polyunsaturated fatty acids 6 and 3, driving an inflammatory process (Liu et al., 2021c). During PM_2.5_-induced inflammation, the gastrointestinal system produces chemokines and leukocytes associated with the pathogenesis of allergic mucosal inflammation. In addition, the generation of nitric oxide by chronic inflammatory processes induces the production of several vasoconstrictors, such as endothelin 1, which is related to the gastrointestinal mucosa and endothelial injury (Liang et al., 2021).

### 3.4 Central nervous system

The damage to the respiratory and cardiac systems due to the infiltration of PM_2.5_ into the bloodstream has been associated with systemic damage (Feng et al., 2016). However, the mechanisms by which PM_2.5_ generates neurotoxicity are still unclear. Initially, PM_2.5_ in the brain must enter the central nervous system (CNS), initiate and then propagate neuronal damage (Zhu et al., 2020). These routes of entry include peripheral inflammation, blood-brain barrier (BBB) damage, and entry through the olfactory bulb or through the gut-brain microbiota axis.

The accumulation of polluting material and PM_2.5_ in the nasal cilia and in the alveoli, after long periods, damages the alveoli therefore inducing lung inflammation by increased expression and release of interleukin 1β (IL-1β), IL-6, and tumor necrosis factor-alpha (TNFα) in lung tissue. This inflammation is expanded towards the heart, kidneys and neuronal tissue, among others (Chen et al., 2017b). However, proinflammatory cytokines released by PM_2.5_ exposure can cause a rupture of the BBB and increased permeability, with a subsequent infiltration of immune cells and proinflammatory cytokines (Kempuraj et al., 2017). BBB rupture, which is a semi-permeable and highly selective barrier that limits the passage of ^s^ubstances from the peripheral circulation to the brain, is achieved because of tight PM_2.5_ interactions with endothelial capillaries, astrocytes, pericytes and neurons. This process in turn allows the entry of neurotoxic substances. After 5 months of exposure to environmental contaminants, great damage to the tight function of the BBB in the CA1 area of the hippocampus was reported (Woodward et al., 2018). Similar damage with increased MMP-2 and BBB permeability was found in ApoE^−/−^ mice exposed to atmospheric pollutants (Oppenheim et al., 2013). In addition, exposure to PM_2.5_ was shown to increase BBB permeability and decrease neuronal viability via MAP2 antigen, macrophage activation, local glutamate increase and ultimately neurotoxicity (Liu et al., 2015a; Liu et al., 2019).

One of the routes of entry of ultrafine particles into the CNS is through the basal membrane of the olfactory bulb (Price and Blakemore, 1985; Calderón-Garcidueñas et al., 2010). The olfactory nerve is the first neural contact after PM_2.5_ inhalation, hence, dendrites of olfactory neurons degenerate upon exposure (Cheng et al., 2016). Previous work has shown that exposure to pollution causes inflammation of the olfactory bulb (Calderón-Garcidueñas et al., 2010) and elevated levels of TNFα in the brain (Chen et al., 2017b). Cellular damage generates hippocampal damage with memory impairment due to the bidirectional axis between the brain and the sympathetic, parasympathetic and enteric systems, being a communication route between these systems (Dinan and Cryan, 2017). Release of pro-inflammatory factors within the brain-intestine imbalance generates cognitive damage and amyloidosis (Mancuso and Santangelo, 2018). Moreover, it was shown that the presence of *IL-6* and mitochondrial ROS in the intestine can reach the CNS and generate damage in the presence of particulate matter (Mutlu et al., 2011; Javurek et al., 2017), which provides further evidence of the relationship between PM and dysbiosis with a direct neuronal damage effect.

#### 3.4.1 Mechanisms of neurotoxicity by PM_2.5_

Upon entry to the CNS, PM_2.5_ triggers a series of neurotoxic events such as neuroinflammation, excitotoxicity, oxidative stress with mitochondrial damage, apoptosis, synapse dysfunction, and neurodegeneration (Kang et al., 2021). Such damage generated by PM_2.5_ impacts different regions of the CNS thereby affecting cognitive, motor, and affective processes (Ku et al., 2016; Peters et al., 2019). During neuroinflammation, a defense response of the host tissue against toxic events begins. However, continuous inflammatory stimulation causes progressive neuronal and neurodegenerative damage (Heppner et al., 2015). Overactivation of microglia and glia releases proinflammatory factors and increases *Gfap* and *Iba-1* expression (Andrade-Oliva et al., 2018). Furthermore, the overactivation of the immune system in the CNS activates the Aβ oligomers clearance in Alzheimer’s animal models (Yang, 2019).

When PM_2.5_ reaches the hippocampus and activates glia and microglia, neuroinflammatory damage is generated. In a nanoparticle exposure study, the release of inflammatory chemicals in the corpus callosum with a consequent increase in IL-1β, IL-6 and TNFα was shown (Babadjouni et al., 2017; Ku et al., 2017). Additionally, changes in the expression of DNA methyltransferase 1 (*DNMT1*) and transcripts related to the JAK2/STAT3, and cytokine pathways were observed (Zhang et al., 2019). Other groups have shown that PM_2.5_ increases the production of glutaminase and glutamate in macrophages and microglia in a reversible manner by the application of an NMDA receptor antagonist and indicating cell damage in an environment of excitotoxicity in the CNS (Liu et al., 2015a).

Exposure to PM_2.5_ has also been linked to other alterations in the hippocampus including cognitive impairment, attention deficit hyperactivity disorder, and autism (Chao et al., 2017; Liu et al., 2021d). Within this context, some authors have reported that fetal exposure to PM_2.5_ promotes a deficit in normal brain development, decreasing neuronal viability and promoting apoptosis by mechanisms dependent on *PKA/CREB/BDNF* signaling deficiency (Zhang et al., 2020a). Another major pathway responsive to PM_2.5_ with transcriptional effects are protein kinase A (PKA) signaling and its downstream effectors, such as cAMP response element binding protein (CREB) (Zhang et al., 2020a). One of the main products of this pathway is brain-derived neurotrophic factor (BDNF), a neurotrophin that binds to its receptors with tyrosine kinase activity and has a very important role in neuronal survival but also participates in the processes of synaptic plasticity and the physiological and morphological processes associated with memory (von Bohlen Und Halbach and von Bohlen Und Halbach, 2018).

Alterations in miRNA expression in the hippocampus and cerebral cortex have been reported that may affect neuronal function and reduce cognitive and motor skills during both the fetal and adult stages. In a study by Chao *et. al.,* pregnant rats exposed to PM_2.5_ displayed an aberrant immune cell activation, with cytokines and free radicals’ accumulation in the amniotic fluid, by an epigenetic mechanism dependent on a miRNA signature (Chao et al., 2017). This signature included upregulated *miR-6315*, *miR-3588*, *miR-466b-5p*, *miR-3560*, and *let-7b-5p*; and downregulated *miR-338-5p*, *let-7e-5p*, *miR-99b-5p*, *miR-92b-5p*, and *miR-99a-5p*; in cerebral cortex and hippocampus at E18 stage, with neural development implications by altering the expression of *Pkn2*, *Gorab*, *Mobp*, *Oxct1*, *Lin28b*, *Kbtbd8* and *Adam11* (Heppner et al., 2015). Furthermore, Ku *et. al.,* demonstrated the detrimental effects of PM_2.5_ aspiration on cognitive function by *miR-574-5p* decrease-mediated activation of BACE1, which induced neuroinflammation and impaired synaptic function (Ku et al., 2017).

## 4 PM-induced DNA and histone modifications within the context of disease-related transcriptional programs

Among the epigenetic mechanisms involved, three main groups are known, including DNA methylation, post-translational histone modification (PTM), and non-coding RNAs (ncRNA). These mechanisms impact gene activity by acting at different levels of the genetic code: at the level of chromatin packaging, at the pre-transcriptional level as marks that allow or inhibit transcription, and at the post-transcriptional level modulating protein translation (O'Brien et al., 2018).

### 4.1 Inflammation

Among the severe inflammatory conditions, rhinitis develops because of a first exposure to PM_2.5_ (Qing et al., 2019). Researchers have found that these particles raise oxidative stress and the production of inflammatory cytokines and chemokines. They also change gene expression and protein secretion in the mucosa and endothelial tissue of the respiratory system in both humans and mice (Wyatt et al., 2020). Short- and long-term exposure to PM_2.5_ promotes the release of IL-6 and IL-8, exacerbating the inflammatory process and producing chronic conditions such as asthma and pulmonary disease (Valderrama et al., 2022). The mild inflammation associated to PM_2.5_ and PM_10_ exposure involves increased levels of biomarkers associated with cardiovascular diseases, such as C-reactive protein (CRP), soluble intercellular adhesion molecule-1 *(*sICAM-1), soluble vascular cell adhesion molecule-1 (sVCAM-1), LDH, propidium iodide, ROS, IL-8, peptidyl arginine deiminase 4 (PAD4), toll-like receptor 4 (TLR4) myeloperoxidase (MPO), and neutrophil elastase (NE) (Valderrama et al., 2022). Despite inflammatory process appear to vary depending on the specific organ or tissue involved, they are generally linked to various pathways regulated by nuclear factor E2-related factor 2 and/or nuclear factor Kappa B, as well as enzymatic activities, including COX-2, iNOS, CRP, CXCR-4 lipoxygenases, IL-1, IL-6, and IL-12. In the cardiovascular system, the inflammatory response induced by OM produces a vasodilation disorder associated with an increase in the expression of *IL8*, *NETs*, *MPO,* and *PAD4* (Xu et al., 2022b). At the vascular level, the inflammatory response is accompanied by an increased expression of *Il6*, TNF type- 2 receptor (*Tnfr2*), *Il1β*, *Il18,* inactive rhomboid-like protein 2 (*iRhom2*) and TNF-α converting enzyme (*Tace*) in murine RAW26_4.7_ cells. Additionally, this upregulation affects the expression of *Iκ-Bα*/nuclear factor kappa-B (*Nf-κB*) and *Tace*/*Tnfr* (Fu et al., 2020; Tang et al., 2021).

**FIGURE 2:**
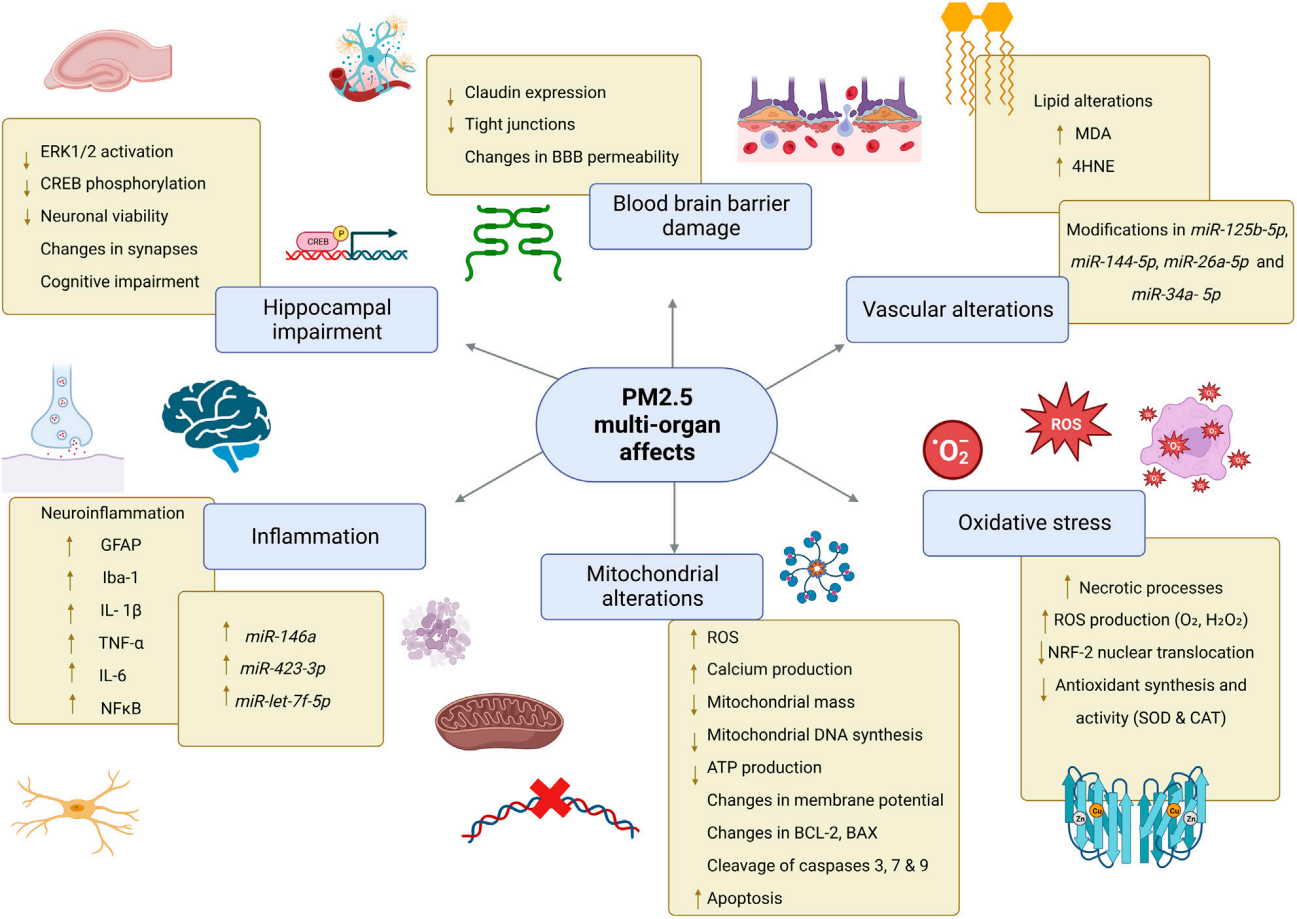
PM_2.5_ multi-organ effects. Exposure to PM_2.5_ promotes alterations in mitochondria which in turn are associated with increased production of reactive oxygen species causing oxidative stress, lipid peroxidation, and increased apoptosis. In the BBB, claudin expression and tight junction formation are reduced, impairing the integrity of this barrier. In the hippocampus PM_2.5_ promotes cell damage that alters neuronal communication and causes cognitive impairment. In different tissues, PM induce differential expression of miRNAs. Abbreviations: PM_2.5_, particulate matter 2.5; CNS, Central Nervous System; mtDNA, mitochondrial DNA; ATP, Adenosine triphosphate; O_2_・, superoxide radical; H_2_O_2_, hydrogen peroxide; SOD, superoxide dismutase; CAT, catalase; MDA, Malondialdehyde; 4-HNE, 4-hydroxy-2-nonenal; BBB, blood-brain barrier; ERK 1/2 Extracellular signal-regulated kinases 1/2; CREB, cAMP response element-binding protein; GFAP, glial fibrillary acidic protein; Iba-1 ionized calcium-binding adapter molecule; *NF-kB*, nuclear factor kappa B; TNF-α tumor necrosis factor alpha; IL-1β, interleukin 1 beta; IL-6, interleukin 6; BCL-2, B cell lymphoma 2; BAX, Bcl-2 Associated X-protein.

Levels of oxidative stress-related DNA damage increase in vascular tissue potentially because of the massive ROS attack on mitochondrial DNA (Figure 2), which has imprinted control regions (ICRs) in imprinted genes and represents a promising biomarker of environmental exposure. For instance, short-term exposure to PM_2.5_ components, specifically transition metals, induced changes in the imprinted gene *RB1,* which correlated with malignant features in non-small cell lung cancer, glioma, and bladder cancer (Liang et al., 2021). In a similar context, the regulation of the *NKX2-3* on inflammatory bowel diseases was examined through cDNA microarrays in individuals exposed to PM_2.5_. Findings revealed a diminished expression of *NKX2-3* contributing to the pathogenesis and progression in inflammatory bowel diseases, largely attributed to the ED1 pathway (Chen et al., 2010). Indeed, inflammatory bowel disease (IBD) increases the risk of colorectal cancer when methyl groups are covalently bound to cytosines in the CpG islands in stool DNA, specifically in loci containing *BMP3*, *VIM, EYA4* and *DRG4* genes (Kisiel and Ahlquist, 2013). In recent years, the correlation between PM_2.5_ exposure and miRNA expression has been proven to play crucial roles in epigenetic regulation controlling inflammation, thrombogenicity, endothelial dysfunction, pulmonary macrophage-epithelial cell crosstalk, and oxidative stress. Circulating inflammatory cells from myocardial infarction patients exposed to PM_2.5_ displayed a miRNA expression profile consistently characterized by upregulated *miR-146a*, a cytokine-responsive miRNA induced by TNF-α and interleukin-1β, *miR-423-3p* and *miR-let-7f-5p* (Cecconi et al., 2022). Data obtained from human and animal models suggest that altered miRNA expression produces the inflammatory microenvironment associated with short-term exposure to PM_2.5_, such as downregulation of PPP2R5C (Protein Phosphatase 2 Regulatory Subunit B ′ Gamma) and the consequent PI3K-Akt signaling pathway inhibition, altering alveolar macrophage proliferation. Moreover, *miR-30c-2–3p* downregulation was shown to correlate with increased PPP2R5C, and *miR-467c-5p* with PRDX6 in a PM_2.5_‐dependent manner, leading to macrophage proliferation and a local inflammatory microenvironment (Liu et al., 2022b).

### 4.2 Oxidative stress

In 1985, Helmut Sies defined oxidative stress as “an alteration in the pro-oxidant/antioxidant balance in favor of the first, which can cause potential damage”. Several updates to this definition have been made to differentiate between harmful and normal levels of oxidative species (Sies, 2020). Oxidative stress has been defined as a transient or long-term increase in oxidant species that causes alterations in metabolic and cell signaling pathways leading to modifications in macromolecules which, if not counteracted, lead the cell to apoptotic or necrotic processes (Lushchak and Storey, 2021). Thus, it is essential to highlight the role of free radicals in oxidative stress. Free radicals, possessing unpaired electrons in their outer orbitals, reactively affect organisms by oxidizing or reducing atoms in proteins, lipids, nucleic acids, and carbohydrates (Forman and Zhang, 2021), earning them the label of reactive species (RS). The Krebs cycle, oxidative phosphorylation, and glycolysis are the metabolic processes that cause mitochondria to produce the majority of RS. During oxidative phosphorylation, electrons pass along complexes composed of molecules that can alternate between oxidized and reduced states, they accept and give electrons away to generate an electron flux, with the final aim of energy production. RS are thus produced due to the natural electron leakage throughout the different complexes of the transport chain (Schieber and Chandel, 2014; Zorov et al., 2014). They are common in biological systems and their presence is crucial to maintain homeostasis. However, modifications in the redox system entail a deleterious process that elicits stress in organisms (Hennig, 1987; Angelova and Abramov, 2018). In this context, it becomes important to emphasize the role of antioxidants which serve as the body’s defense mechanism against reactive species. Antioxidants are enzymes that play an important role in the prevention and reduction of free radical-induced damage to macromolecules (Halliwell and M C Gutteridge, 2022). Several factors affect oxidative stress, such as nutrition, aging, and response to pathogens, modulating the increase of reactive species or the decrease of antioxidant enzymes (Briones-Martin-Del-Campo et al., 2014; Zhang et al., 2015; Liu et al., 2018; Tan et al., 2018). In addition to mitochondria, exogenous oxidative sources to which organisms are exposed can become direct oxidants such as pollutants found in the environment that affect the molecular expression of epigenetic factors (Briones-Martin-Del-Campo et al., 2014). Research has shown that PM_2.5_ produced by air pollution has a high oxidizing potential (Wells and Zelcer, 1986; Caumo et al., 2023; Liu et al., 2023). Because PM_2.5_ particles are mainly composed of copper, iron, secondary aerosols, and quinones, different studies have shown the oxidative potential of PM_2.5_ in lung and cardiac cells, as they increase the levels of reactive oxygen species mainly superoxide anion (O_2_^•-^), hydroxyl radical (OH^•^) and hydrogen peroxide (H_2_O_2_) while decreasing the synthesis and activity of antioxidant enzymes such as superoxide dismutases (SOD) (Lelieveld et al., 2021; Lin et al., 2022; Zou et al., 2022).

#### 4.2.1 Impact of PM_2.5_ in mitochondria

The effects of PM_2.5_ exposure on mitochondria have been extensively studied. *In vitro* models have reported that the main effects include a decrease in mitochondrial mass, mitochondrial DNA synthesis, as well as a reduction in cellular respiration (Sivakumar and Kurian, 2023; Li et al., 2022). These effects are believed to be caused by the opening of the mitochondrial permeability transition pore, resulting in excessive calcium production and changes in the mitochondrial membrane potential (Zou et al., 2022). The mitochondrial membrane potential is driven by oxidation-reduction reactions associated with Krebs cycle activity and oxidative phosphorylation, which forms an electrochemical gradient that ultimately promotes ATP synthase activity towards ATP generation (Zorova et al., 2018). Changes in membrane potential are associated with mitochondrial damage, then any increase in mitochondrial membrane potential leads to ROS generation (Li et al., 2022). Several studies have reported that exposure to PM_2.5_ results in a decrease in mitochondrial membrane potential and ATP production (Esteras et al., 2017; Zorova et al., 2018; RRCui et al., 2020; Gao et al., 2022). One of the main ROS produced is the superoxide anion, which is generated by oxidative phosphorylation in mitochondria. This radical is generated by the leakage of electrons in the respiratory chain and its reaction with oxygen (Kim and Keum, 2016). Manganese SOD (MnSOD, SOD2) and copper/zinc SOD (Cu/ZnSOD, SOD1) convert O_2_^•-^ into H_2_O_2_ which in turn is metabolized by catalase (CAT) into water molecules (Alfonso-Prieto et al., 2009; Azadmanesh and Borgstahl, 2018; Wang et al., 2018; Rosa et al., 2021). Moreover, exposure to environmental PM_2.5_ reduces the expression and activity of the antioxidant enzymes SOD2 and CAT and hence enhances the production of intracellular and mitochondrial ROS (Li et al., 2013; RRCui et al., 2020; Zhai et al., 2022). Moreover, some reports indicated changes in the expression of *Bcl-2* (B cell lymphoma 2) and *BAX* (Bcl-2 Associated X-Protein) and the activation of the pro-apoptotic caspases 3, 7 and 9 in cell cultures exposed to PM_2.5_ (Wei et al., 2021; Lin et al., 2022). BAX is a cytosolic protein that is controlled by the tumor suppressor protein TP53 (Huska et al., 2019; Pisani et al., 2020). Other authors have reported that changes in *BAX* expression in environments with high PM_2.5_ concentrations is a direct consequence of TP53 phosphorylation at serine 115 (Ser115) (Wang et al., 2017). The activity of TP53 on *BAX* allows the opening of a pore in the mitochondrial membrane and, consequently, the release of cytochrome C which binds the protein APAF1 (Apoptosis protease-activating factor-1). In consequence, APAF1 initiates the formation of the apoptosome, a protein complex that mediates the caspase 3 activation and ultimately promotes the activity of CAD (Caspase-activated DNase) which fragments DNA and ultimately leads to cell death by apoptosis (Chipuk et al., 2004; Ulukaya et al., 2011; Umamaheswari et al., 2021). On the other hand, it is known that nuclear factor erythroid 2-related factor 2 (NRF2) can alter the level of methylation of CpG islands in the promoter of the cytochrome *P450* gene, an antioxidant enzyme whose expression has been implicated in the generation of lung injury (Wu et al., 2023a).

NRF2 is a transcription factor that governs the expression of antioxidant enzymes and is anchored to KEAP1, which negatively regulates its activity. Under normal states, NRF2 is susceptible to modifications by ROS, such modifications occur at cysteine residues and allow NRF2 to dissociate from KEAP1 and translocate to the nucleus where it upregulates the transcription of genes containing antioxidant response elements (ARE) in their promoter region (Ahmed et al., 2017; Saha et al., 2020). However, NRF2 is inactivated in some pathological conditions, which prevents the synthesis of antioxidant enzymes (Kasai et al., 2020). In this regard, repeated exposure to particulate matter was reported to increase cytoplasmic levels of NRF2, without translocation of NRF2 to the nucleus, resulting in reduced synthesis of antioxidant enzymes (Orona et al., 2019).

#### 4.2.2 Interplay between PM_2.5_ and lipoperoxidation

Several reports have indicated a close and functional association between lipid peroxidation and PM_2.5_ damage due to mitochondrial dysfunction. Lipids are essential for physiological processes including membrane maintenance, energy production, and cell signaling. Yet alterations in lipid homeostasis favor the appearance of pathological changes that contribute to the development of diseases (Ademowo et al., 2017; Angelova et al., 2021). Lipid peroxidation is a process in which oxidizing agents (such as free radicals) react with lipids containing one or more carbon-carbon double bonds and generate toxic products that affect cell integrity, such as malondialdehyde (MDA) and 4-hydroxy-2-nonenal (4-HNE) (Zanetti et al., 1988; Ayala et al., 2014). In this regard, exposure to PM_2.5_ has been reported to result in increased production of MDA in human bronchial epithelium cultures (Zhai et al., 2022) and in human urine and serum samples (Zhang et al., 2020b; Fan et al., 2023). Increased 4-HNE expression has also been shown in mouse brain tissues (Piao et al., 2018). However, the precise means by which these toxicants affect antioxidant capacity and mitochondrial activity in environments with high exposure to PM_2.5_ particulate matter remains to be elucidated. In conclusion, considerable evidence defines environmental pollution, especially where there is a high presence of PM_2.5_ particulate matter, as an important source of inducing changes in mitochondria that promote a state of oxidative stress and apoptotic processes in various cell types. Oxidative stress is a very important factor that predisposes to the development of multiple diseases, including pulmonary, cardiovascular, renal, and metabolic diseases and neurodegenerative disorders.

### 4.3 Cancer

Understanding most cancer-associated diseases and the impact of air pollution requires a multifactorial approach that addresses genetic, epigenetic, stochastic, and environmental factors. The risk of lung cancer is positively correlated with the levels of air pollution since regions with a high level of pollution present an increase in mortality (Loomis et al., 2013; Brauer et al., 2016; Real et al., 2021). Particulate matter and its impact on human health are currently being extensively investigated. An study carried out in the years 2004–2008 on inhabitants of the Augsburg region of southern Germany aged 25–74 years old on short- and medium-term exposure to PM_2.5_, using human methylation arrays data identified several significant CpG sites that have adverse health effects through variations in DNA methylation (Panni et al., 2016). This data is the result of a collection of phases in the long-term KORA cohort studies in Germany, focused on uncovering health, risk factors, and chronic diseases in the Augsburg region’s population, providing valuable insights into public health and epidemiology. Specific cohort studies based on EWAS revealed several unique CpG island sites previously unassociated with air pollution such as the cg03513315 CpG site located in the PES1 (Pescadillo Ribosomal Biogenesis Factor) gene positively associated with PM_10_ and PM_2.5_ exposure in the European Prospective Investigation into Cancer and Nutrition-The Netherlands (EPIC-NL). These results suggested that *PES1* plays a role in proliferation and tumorigenesis in breast cancer (Holle et al., 2005; Wichmann et al., 2005).

The effect of polychlorinated biphenyls (PCBs) has been analyzed on the risk of prostate cancer and metastasis risk, observing that exposure to dioxins and PCB-153 (di-ortho-2,2′, 4, 4′, 5, 5′-hexachlorophenyl) promotes an aggressive state and accelerates mitosis progression. In this context, pharmacological and genetic inhibition of *ACAT1* (microsomal enzyme acetyl-coenzyme A-acetyl transferase 1) has been proven to attenuate tumor growth. Bunay *et. al*., (Buñay et al., 2023), showed that dioxins and PCB-153 induced the upregulation of *ACAT1* through the activation of AHR (aryl hydrocarbon receptors), which was associated with an aggressive phenotype and increased tumor growth, migration, cellular invasion and metastatic progression of prostate cancer (Buñay et al., 2023).

DNA double-strand breaks and adducts induced by air pollution showed a strong link to cancer and repair capability influencing adduct removal (Demetriou and Vineis, 2015). Both the *TP53* mutation and inactivation contribute to the pathogenesis of lung cancer (Deben et al., 2017). PM_2.5_ at low doses was able to induce epigenetic silencing of *TP53* in human alveolar epithelial cells (Zhou et al., 2016). Remarkably, the number of mutations caused was three times higher in lung cancers related to air pollution than in lung cancers from low-exposed regions (Turner et al., 2020). Moreover, *TP53* hypermethylation contributes to gene silencing, while DNA hypomethylation causes chromosomal instability and activation of retrotransposons and repetitive elements such as *LINE-1* and *Alu*. DNA hypomethylation also affects critical chromosomal regions, such as subtelomeric regions and pericentromeric regions, in both short- and long-term manners as proven in polluted regions in China, Northern Italy and the Northeastern United States of America (Turner et al., 2020).

Mechanistically, long-term exposure of lung cancer cells to PM_2.5_ may promote lung cancer progression through activation of the Aryl hydrocarbon receptor (AhR) and epidermal growth factor receptor (EGFR), which boosts the Serine protease pathway transmembrane 2 (TMPRSS2)-IL8 pathway. *STC2* induced by PM_2.5_ could be a potential immunological biomarker not only for the prognosis of lung cancer but also additional cancers related to air pollution (Zhu et al., 2023). It has also been shown that human epithelial cells exposed to PM_2.5_ are more susceptible to hypomethylation and transcriptional activation of several coding genes as well as microRNA, which induces modified signaling pathways related to cancer (Heßelbach et al., 2017). PM_2.5_ can also induce changes in long non-coding RNAs, such as *LOC146880*, through ROS, promoting autophagy and malignancy in lung cells (Deng et al., 2017).

Transcriptional changes in miRNA of human bronchial cells exposed to environmental PM_2.5_ have also been described, including downregulation of *miR-182* and *miR-185*, potentially downregulating oncogenes (*SLC30A1*, *SERPINB2*, and *AKR1C1*) and facilitating neoplastic transformation (Liu et al., 2015b). A positive association has been observed between PM_2.5_ exposure and BPDE (benzo[a]pyrene 7,8-diol-9,10-epoxide)-induced DNA adducts (Li et al., 2014). Another study in the Chinese population suggested season-related genotoxic activity of PM_2.5_ in human bronchial epithelial cells (16HBE), showing a greater increase in oxidative damage to DNA and DSB in winter due to emissions from industrial boilers and heating boilers, which create unique air pollutants (Niu et al., 2020). Therefore, genetic damage could be the potential biological mechanism of carcinogenesis derived from air pollution, and the activation of DNA repair factors could be essential to overcome this process. Exposure to high levels of PM_2.5_ has been shown to inhibit the function of the homologous recombination repair (HRR) pathway by increasing *RAD51* methylation, leading to the inability to repair DNA double-strand breaks (DSBs). *MGMT* or *ERCC1* methylation in the blood could be used as sensitive biomarkers for rectal tumors (Shalaby et al., 2018). Another important obervation is the arrest of the cell cycle in the G2/M phase when exposing 16HBE cells to PM_2.5_, a mechanism mediated by the expression of the lncRNA *LINC00341* and suppression of *p21* (Xu et al., 2017). Alterations in glycolysis/gluconeogenesis suppressed genome instability*;* thus*,* differential expression of miRNA in lung tissues was associated with DNA damage, cell cycle, and apoptotic genetic damage repair, which could play an important role in the cancer process (Hanasaki et al., 2020).

### 4.4 Fibrosis

Fibrosis is a medical condition characterized by the excessive accumulation of ECM components (such as collagen and fibronectin) because of aberrant injury repair processes and a lower ECM degradation rate. This debilitating condition leads to organ dysfunction and failure by altering the oxygen supply (Wynn and Ramalingam, 2012). Depending on the tissue, molecular characteristics, and grade of organ dysfunction, fibrosis is considered a life-threatening condition. Renal, cardiac, liver and pulmonary fibrosis are the major causes of death, but several studies have identified common altered epigenetic regulation that could be a potential approach to target multi-organ fibrosis (Rubio et al., 2023b). Particularly, pulmonary fibrosis (PF) is the irreversible phenotype of all interstitial lung diseases (ILDs), characterized by progressive thickening of lung tissue that reduces gas exchange and ultimately leads to progressive hypoxemia, respiratory failure, and mortality (Zhu et al., 2023). PF is an age-related ILD characterized by a failure in lung alveolar epithelial regeneration and an abnormal wound-healing response (Chanda et al., 2019). Ambient air pollution is increasingly recognized as a risk factor since the progress, the incidence and prognosis of PF have been associated with occupational metal and wood dust exposures as well as long-term personal exposure to air pollutants, independently of age, gender, smoking history, lung function, and antifibrotic treatment (Johannson et al., 2015; Tomos et al., 2021; Yoon et al., 2021; Cui et al., 2023). Specifically, the potential mechanisms by which organic components of PM could cause, exacerbate, or accelerate fibrogenesis could be via oxidative stress and other profibrotic signals inducing cellular senescence, dysregulated fibrogenesis, and/or inflammation leading to epithelial damage and fibrosis (Johannson et al., 2015; Tomos et al., 2021). Despite the increasing population-based and epidemiologic associations between PM exposure and PF development, little is known about the impact of PM on the epigenome of PF patients, even though some recent efforts have interrogated epigenomic alterations in different PF experimental approaches (Rubio et al., 2019; Goobie et al., 2020; Dobersch et al., 2021; Díaz-Piña et al., 2022).

Exposure to PM might be associated with an increased risk of PF, possibly through its effect on inflammatory response regulation, in which some miRNAs are among the main epigenetic regulators. For instance, *miR-155* is highly expressed in lung epithelial cells and circulating neutrophils in cystic fibrosis (CF). This may contribute to the expression of the proinflammatory *IL-8* in CF by lowering *SHIP1* (SH2-containing inositol-5′-phosphatase 1) expression, which is a regulator of inflammation and an activator of the PI3K/Akt signaling pathway (Bhattacharyya et al., 2011). Moreover, *miR-155-5p* was reported to mediate the associations between benzo[a]pyrene-r-7, t-8, t-9, and c-10-tetrahydro tetrol-albumin (BPDE-Alb) adducts (an internal exposure biomarker of PM) and *IL-6* and/or *TLR2* expression in a pediatric population highly exposed to PM, (Li et al., 2020a). In a similar manner, *miR-6238* is specifically upregulated in lung tissue and lung-derived extracellular vesicles (EVs) in response to PM exposure. This miRNA is in turn internalized into alveolar macrophages (AMs), resulting in neutrophil infiltration of lung alveoli and pulmonary inflammation (Park et al., 2023). As previously shown, miRNA dysregulation induced by PM_2.5_ is one of the potential mechanisms of pulmonary inflammation. It was found on human bronchial epithelial cells (HBECs) exposed to PM_2.5_ that downregulation of *miR-382-5p* exacerbates the inflammatory response through elevated *CXCL12* (a cytokine also known as stromal cell-derived factor-1, SDF-1) and *MMP9* (metalloproteinase-9). The later mechanism also influences eosinophil trafficking and correlates with (Zhang et al., 2020c). In particular, PM_10_ may also dysregulate miRNA expression as shown in vitro assays. Exposing cells to PM_10_ for 48 h reduces *miR-26a* expression, boosting epithelial-mesenchymal transition (EMT) via IL6 and STAT3 activation (Lu et al., 2018). Similarly, STAT3 activation can stimulate fibroblast-to-myofibroblast shift, collagen release, and fibrosis through *SOCS3* hypermethylation, induced by TGFβ as evidenced *in vivo* (Dees et al., 2020).

Genome-wide DNA methylation and RNA-transcription analysis in bronchial epithelial cells (BEAS-2B) found 6,835 differentially methylated CpG. Most of the changes corresponded to hypomethylation (68.93%), 50.36% of them were present in promoter regions of genes that are mostly linked to respiratory insufficiency, pneumonia, fibrosis, lung cancer, and other diseases (Shi et al., 2019). In peripheral blood samples of patients with Idiopathic Pulmonary Fibrosis (IPF), Goobie *et al.* described that increased long-term PM_2.5_ exposure correlated with higher global DNA methylation (Goobie et al., 2023). In another genome-wide study Huang *et al.* further evaluated how different concentrations and durations (single or repeated doses) of PM_2.5_ exposure differentially affect both the BEAS-2B transcriptome and DNA methylome (Huang et al., 2021). For instance, a single treatment of high-concentrated PM_2.5_ (30 µg/cm^2^) for 24 h showed a slight tendency towards global hypomethylation across the genome. However, repeated exposure to low-concentrated PM_2.5_ (1 µg/cm^2^) for 7 days caused a more noticeable degree of hypomethylation across the genome linked to an increase in the expression of the-ten–eleven translocation (TET) enzymes (*TET1*, *TET2*, and *TET3*) which participate in the demethylation mechanism (Huang et al., 2021).

Ju *et al.* additionally compared a higher dose of 100 μg/ml *versus* 0 and 50 μg/ml PM_2.5_ unexposed human bronchial epithelial (16HBE) cells. Interestingly, these authors identified that altered N6-methyladenosine (m6A) modification of nuclear factor E2-related factor 2 (*NRF2*) mRNA enhanced its translation and affected the development of PF by binding the m6A-binding proteins, such as YTH domain-containing proteins (YTHDFs) and insulin-like growth factor 2 mRNA-binding proteins (IGF2BPs) (Ji et al., 2023). Similarly, Ning *et al.* evaluated equivalent doses in BEAS-2B cells and found that PM_2.5_ treatment downregulated E-cadherin and upregulated vimentin at both protein and mRNA levels in a dose-response manner (Ning et al., 2022).

The EMT process involves some signal transduction pathways, such as the yes-associated protein 1 (YAP1), Wingless/Int (WNT), and TGF-β that are linked to the pathogenesis of fibrosis (Piersma et al., 2015). TGF-β, considered an important regulatory center in the IPF epithelial network, promotes the fibrotic process via multiple signaling pathways, including the Smad, MAPK, and ERK signaling pathways, as well as epigenetic alterations (Ye and Hu, 2021). In this regard, PM_2.5_ exposure increases N-acetyltransferase 10 (NAT10) levels, which catalyze *TGFB1* N4-acetylcytidine (ac4C) modification, which enhances its stability and ultimately accelerates lung EMT and fibrosis (Wu et al., 2023b). Furthermore, chronic PM_2.5_ exposure could not only directly trigger activation of pulmonary fibroblasts and EMT in BEAS-2B and in human pulmonary fibroblast (HFL-1), but also indirectly promote fibroblast phenotypic transformation by extracellular signals. Thus, PM_2.5_ could be a potent initiator of PF through TGF-β1 activation (Xu et al., 2019b). Under PM_2.5_ exposure, TGF-β activation might be coordinated by the function of lncRNAs, which are also abnormally expressed in fibrotic diseases (Zhang et al., 2018). One promising candidate may be the maternally expressed gene 3 (*MEG3*), which can regulate the expression of pulmonary airway epithelial cell genes associated with the basal-like state, as shown by single-cell sequencing of pulmonary epithelial cells isolated from IPF lung tissues (Gokey et al., 2018). *MEG3* expression was negatively correlated with normal alveolar type 2 (AT2) epithelial cell gene markers (*STFPC*, *SFTPB*, *SFTPA1*, *SFTPD*, and *NAPSA*) and normal alveolar type 1 (AT1) gene marker (*HOPX*) expression. In contrast, *MEG3* positively correlated with transcripts associated with basal cells such as *TP63*, *ITGB4*, *KRT17*, and KRT5, which regulate epithelial cell differentiation (Gokey et al., 2018). *MEG3* can also promote epithelial damage, according to a study in HBECs cells acutely exposed to PM_2.5_, *MEG3* was substantially upregulated, thereby mediating cell apoptosis and autophagy by increasing the expression of the tumor suppressor *TP53* in a reversible manner (Li et al., 2018). Consequently, *MEG3* may be a potential therapeutic target to overcome PM-dependent injuries. Finally, evidence obtained in carbon tetrachloride (CCl4)-induced human fibrotic livers showed that *MEG3* levels were significantly reduced (He et al., 2014), indicating that the potential role of *MEG3* in fibrosis may differ between cell types and human organs.

### 4.5 Development

**FIGURE 3:**
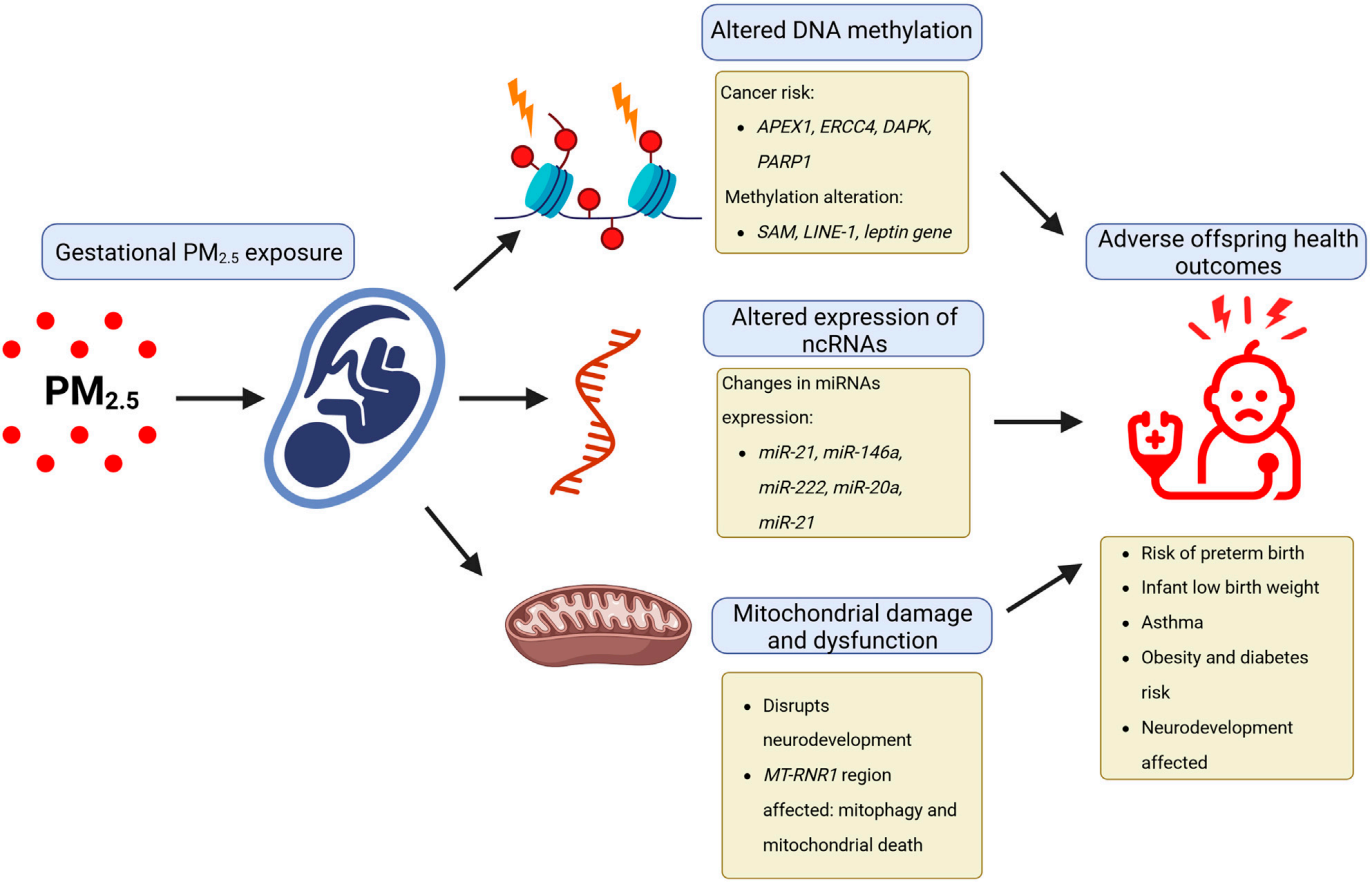
Epigenetic effects of gestational PM_2.5_ exposure affect human development. The negative impact of environmental pollutants, such as particulate matter with a diameter of less than 2.5 μm (PM_2.5_) during pregnancy, can affect fetal development and long-term child health through epigenetic effects caused by altered expression of ncRNAs, altered DNA methylation, and mitochondrial damage.

As urbanization continues around the world, more pregnant women are exposed to environmental hazards such as air pollution and, at the same time, have less contact with natural environments (Zanini et al., 2020). These hazards can negatively impact both the mother and the developing child because pregnancy is a time of significant physiological and metabolic changes, rendering them vulnerable to these environmental risks (Figure 3) (Zanini et al., 2020). Multiple evidence regarding the negative impact of environmental pollutants such as PM_2.5_ during pregnancy has been published, which can significantly impact the development of fetuses and children’s long-term health (Feng et al., 2016; Bekkar et al., 2020; Johnson et al., 2021; Pryor et al., 2022; Thangavel et al., 2022; Kaur et al., 2023). Increasing evidence from human cohort studies indicates that exposure to fine PM, like PM_2.5_, during pregnancy triggers a series of interrelated pathological processes through chemical modifications to DNA, particularly methylation, as well as non-coding RNA dysregulation (Johnson et al., 2021).

DNA methylation is extensively studied as one of the key epigenetic modifications that links prenatal exposure to PM_2.5_ with adverse health outcomes in offspring (Vaiserman, 2015)^**.**^ For example, the placenta is affected during gestation by PM_2.5_ exposure in mothers, altered DNA methylation occurs, including global hypomethylation as well as specific gene-related methylation changes (Janssen et al., 2013). Hypermethylation, on the other hand, is linked to developmental defects such as gestational diabetes and Down’s syndrome in a different time window of gestational development (Jin et al., 2013; Reichetzeder et al., 2016). Additionally, exposure to PM_2.5_ during the first trimester of pregnancy has been correlated with decreased global DNA methylation in placental tissue (Janssen et al., 2013) and altered gene expression of S-adenosylmethionine (*SAM*), responsible of methyl groups’ transfer (Kim et al., 2021; Maghbooli et al., 2018). Furthermore, significant DNA methylation changes have been reported in promoter regions of genes related to fetal growth and development as a result of maternal PM_2.5_ exposure, such as the leptin gene (*LEP*) (Saenen et al., 2017). It has also been observed that altered DNA methylation in gene promoters in newborns’ blood, some of which were related to cardio-respiratory outcomes during childhood (Breton et al., 2016). Of great interest and concern, maternal PM_2.5_ exposure has been correlated with altered DNA methylation in promoter regions of key DNA repair and tumor suppressor genes, including *APEX1, ERCC4, DAPK, and PARP1*. These findings suggest that changes in fetal and neonatal capacity to repair DNA sequences are altered by PM_2.5_ exposure and may contribute to a later increase in cancer risk (Alvarado-Cruz et al., 2017; Neven et al., 2018).

As an interplay effector between epigenomics and metabolomics, exposure to PM_2.5_ during pregnancy also impacts mitochondrial function. A recent study revealed that the neurodevelopmental effects associated with prenatal PM_2.5_ exposure are partially influenced by long-term alterations in mitochondrial respiration (Frye et al., 2021). Another article reported that *in utero* exposure to PM_2.5_ resulted in lower mitochondrial DNA (mtDNA) content, as well as modified methylation levels in the *MT-RNR1* region, supporting the association between PM_2.5_ exposure and epigenetic changes in placental mtDNA (Janssen et al., 2013). In the context of PM_2.5_ exposure, studies have shown changes in miRNA expression in placental tissues during different trimesters of pregnancy. For example, the expression of *miR-21*, *miR-146a*, and *miR*-222 significantly decreased following PM_2.5_ exposure in the second trimester, while *miR-20a* and *miR-21* increased in response to PM_2.5_ during the first trimester. The above-mentioned miRNAs are involved in vascular cell proliferation, apoptosis regulation, and maintaining lung physiology (Tsamou et al., 2018). Further research is needed to understand the possible long-term health effects of these changes in ncRNA expression, and the potential involvement of other ncRNA biotypes, in response to PM_2.5_ exposure at the gestational level (Zheng et al., 2020; Deyssenroth et al., 2021; Brunst et al., 2022; Enquobahrie et al., 2022; Wu et al., 2023c).

### 4.6 Cardiovascular diseases

Cardiovascular diseases (CVD) are a group of pathologies that affect the heart and blood vessels. They are the leading cause of death worldwide, causing 1_7.9_ million deaths per year. Among the causal factors that can be modified by individuals for the development of these conditions are diet, physical activity, nicotine exposure, sleep duration, body mass index, blood lipids, blood glucose, and blood pressure (American Heart Association, 2023).

In recent years, several epigenetic mechanisms have become robust tools for the study of CVD. As an example, the CARDIA (Coronary Artery Risk Development in Young Adults Study) research study demonstrated that DNA methylation approaches can accurately predict, prevent, and diagnose CVD (Zheng et al., 2022). Specifically, this study analyzed and associated clinical, biochemical, and epigenomic data from 1,085 individuals over 20 years, with comparisons drawn to the Framingham Heart Study (FHS) data. Researchers identified 45 DNA methylation patterns in genes related to lipid metabolism, insulin secretion, and cytokine production pathways. However, three methylation markers located in the *SARS1, SOCS3,* and *LINC-PINT* genes were found to mediate only 20.4% of the differential effect between cardiovascular health and the risk of coronary artery calcification. Consequently, a methylation-based score was generated to assess the risk of coronary artery calcification and CVD (Zheng et al., 2022).

A variety of molecular processes are involved in the pathophysiology of CVD due to PM. However, oxidative stress and inflammation are among the most studied (Bhatnagar, 2022). Endothelial dysfunction, vascular inflammation, adipocyte inflammation, coagulation disorders, autonomic nervous system stimulation, and epigenetic alterations (Al-Kindi et al., 2020) have been reported to be direct effects of PM by altering oxidative stress and inflammatory pathways (Al-Kindi et al., 2020).

In terms of epigenetic regulation, PM deregulates DNA methylation, leading to cardiovascular diseases such as high blood pressure. A recent systematic review found DNA methylation sites associated with CVD and PM_2.5_. CpG sites cgo1656216 (near *ZNF438*) and cgo3636183 (near *F2RL3*) were significantly associated with CVD and air pollution (Krolevets et al., 2023). Similarly, some clinical trials have shown the association between DNA methylation, CVD, and PM. Prunicki *et al.* analyzed exposure to PM_2.5_, along with other environmental toxins, in 221 school-age children. The authors demonstrated that 5- and 6-ring polycyclic aromatic hydrocarbons (PAH456), ozone (O_3_), carbon monoxide (CO), elemental carbon, nitrogen dioxide (NO_2_), oxides of nitrogen (NO_x_), and PM_2.5_ can induce the methylation of numerous CpG sites related to immunoregulatory genes (*IL-4, IL-10, FOXP3* and *IFN*) and induce high blood pressure (Prunicki et al., 2021). These findings have a significant impact on morbidity and mortality since high blood pressure is the main risk factor for CVD-related death (Organización Panamericana de la Salud, 2023). Therefore, PM_2.5_ could induce CVD by altering DNA methylation during development.

Importantly, PM potentially modifies histones with consequent alteration of cardiac structures. For example, previous clinical studies have shown that PM exposure induces histone 3 trimethylation (H3K4me3) in neonates, increased circulating levels of histone 3 (H3) in school-age children, and H3 trimethylation (H3K9me3, H3K27me3 and H3K36me3) and acetylation (H3K9ac) in adults (Zheng et al., 2017; Vrijens et al., 2020; Suhaimi et al., 2021). Remarkably, some of these histone modifications are also associated with CVD. A study by Wu *et al.,* using animal models, demonstrated that PM_2.5_ exposure in pregnant mice increases acetylation in H3K9ac, with associated anatomical damage to cardiac structures in newborn and adult mice (Wu et al., 2019).

It is important to highlight that histones can be modified by oxidative stress (Niu et al., 2015), thus representing one of the mechanisms activated by PM exposure (Li et al., 2008). ncRNAs can change their expression profile in response to exposure to polluted air (Konduracka et al., 2022; Du et al., 2023). In addition, different ncRNAs act as epigenetic biomarkers in different cardiovascular diseases, such as high blood pressure (Jusic et al., 2019) and atherosclerosis (Aryal et al., 2014). ncRNAs studies have diversified towards their association with clinical CVD stages and CVD biomarkers. In the case of research work by Chen *et al.* (Chen et al., 2022), they analyzed the associations between PM, microRNAs, and cardiovascular biomarkers in 24 healthy adults. This study demonstrated that exposure to PM, ozone (O_3_), and nitrogen dioxide (NO_2_) modifies the expression of different circulating microRNAs (*miR-125b-5p, miR-144-5p, miR-26a-5p, and miR-34a-5p,* among others) and it is associated with changes in blood lipids, serum amyloid A (SAA), C-reactive protein, soluble vascular adhesion molecules 1 (sICAM1), total cholesterol, and high-density lipoproteins (HDL). Consistently, Cecconi *et al.*, observed in patients with acute myocardial infarction exposed to PM_2.5_ that the number of CD4^+^ regulatory T cells and the expression of microRNAs such as *miR-146a-5p, miR-423-3p,* and *miR-let-7f-5p* were similarly affected. This finding evidences the usefulness of epigenetic biomarkers as not exclusive of chronic clinical events but also of acute ones (Cecconi et al., 2022).

## 5 Methods for PM analysis

### 5.1 PM environmental monitoring

Quantifying and determining the composition of PM in the environment is essential to assess the magnitude of exposure and the health risk to the population. The WHO has set guidelines for PM_2.5_ and PM_10_ in the atmosphere at 10 and 20 μg/m^3^, respectively, for the annual average concentration; and at 25 and 50 μg/m^3^, respectively, for the daily average (World Health Organization, 2021). To ensure compliance with the air quality standards, ambient PM concentrations must be monitored continually. The traditional approach used to assess PM concentrations is usually based on fixed-location monitoring stations (Dos Santos Souza et al., 2021).

The US Environmental Protection Agency specifies the gravimetric method as the Federal Reference Method (FRM) for PM mass concentration measurement. This method calculates the PM mass concentration by weighing the particles accumulated on a filter over some period (usually 24 h). Although it is a reliable method, it has the disadvantage of not reporting PM concentrations in real-time but rather the accumulated PM mass concentration (Li et al., 2020b).

The main instruments to quantify particulate matter using the gravimetric method are the High-Volume Air samplers (HVS) and Low Volume Air samplers (LVS) (Sugita et al., 2019). The difference between HVS and LVS is the flow rate (amount of air sampled). HVS typically samples more than 1,500 m^3^ of air over 24 h, while LVS draws through only 24 m^3^ of air or less. These types of equipment use quartz or Teflon filters of different diameters for PM sampling. Before the sampling, the filters equilibrated in conditions of <40% relative humidity and 25 °C room temperature for over 48 h and then weighed on a high-precision microbalance (Li et al., 2020b). Baking the filters for 12 h at 550°C before their use in the sampler is advisable to minimize the blank concentrations (Wang et al., 2012). After sampling, the filter is weighed again, and the difference in filter weight is the collected particulate matter mass. Dividing the mass by the volume of air sampled gives the PM concentration. Both samplers have the same performance in terms of measuring the elemental carbon (EC), organic carbon (OC), inorganic ions (Na^+^, NH_4_^+^, K^+^, and SO_4_^2−^), and low-volatile polycyclic aromatic hydrocarbons (PAHs) (Sugita et al., 2019).

Tapered element oscillating microbalance (TEOM) is another technique for measuring PM. The TEOM is based on the fact that the natural frequency of oscillation of a bar (resonance frequency) is a function of its mass. In these samplers, the tapered element consists of a filter cartridge mounted on the tip of a hollow glass tube. The base of the tube cannot move, but the tip is free to vibrate at its natural frequency. An opposing magnetic field maintains the natural oscillation. The additional weight from particles collected on the filter changes the tube’s frequency. The equipment senses this change and calculates the particle mass rate from the magnitude of the frequency change. Dividing the mass rate by the flow rate provides the particle mass concentration (Cyrys et al., 2001).

Particulate matter can also be measured with automatic equipment, such as Beta Attenuation Monitors (BAMs), which allow real-time data to be obtained. BAMs are continuous monitors measuring beta transmission through a C-14 source. These samplers use tape filters (fiberglass or Teflon) that are changed every 2–3 months. The BAM measures the beta transmission in the blank part of the filter, collects the particulate material for 1 hour, and then measures the beta transmission again; the more particles deposited on the tape during the hour, the lower the signal attenuation. The collected PM mass is calculated as the difference in beta transmission between the blank (clean, without PM) and exposed filter (Raja et al., 2017).

Although the WHO has urged countries to monitor air quality continuously, few countries cover most of their territorial area with fixed monitors for air pollution monitoring due to the high costs of installation and maintenance of the equipment (World Health Organization, 2021). Therefore, alternative methods have been proposed for monitoring air quality, including particulate matter. Satellite data and personal monitors are an option for PM assessment.

*PM satellite monitoring*. In most developing regions, there are no PM ground monitoring networks. If available, this monitoring is limited to some areas (only big cities). In these cases, satellite-retrieved aerosol optical depth (AOD) can be used for air pollution monitoring, including PM monitoring. Satellite AOD data with broad spatial coverage, a long data record, and high spatial resolutions could support assessing historic air pollution levels in developing regions (Xiao et al., 2018; Huang et al., 2019). Satellite AOD data has been increasingly used to predict PM concentrations (prospectively or retrospectively), for which the statistical models that integrate meteorological parameters, emission sources, and land use information are required (Hu et al., 2014; Huang et al., 2018). These statistical models must be validated with PM ground monitoring data (Xiao et al., 2018). The satellite AOD prediction models to assess the exposure levels to PM can be used for large-scale epidemiological cohort studies of PM health effects, as they have done in the Global Burden of Diseases study, and in the US Medicare study (Cohen et al., 2017; Di et al., 2017).

*PM personal monitoring*. Environmental monitoring networks are limited to assessing the quality of ambient (outdoor) air, leaving this assessment outside of indoor and public spaces. Since people move in different microenvironments (home, work, school, public transport, *etc.*) daily and spend most of their time within them, using personal monitors is the best way to assess PM exposure (Koehler and Peters, 2015). Personal monitoring instruments have sensors that can quantify the particle mass, the number of particles, particle surface area, and particle size distribution. Measurement methods include gravimetric (impactors, gravimetric filters), optical (photometers, optical particle counter, condensation particle counter), and electrical (diffusion size classifier, nanoparticle surface area monitor). The main advantages are that they are low-cost, portable, and PM monitoring in real-time (Lowther et al., 2019).

### 5.2 PM composition analysis

Identifying and quantifying PM composition is essential for health risk assessment because PM may contain toxic species. Environmental toxicants can interact with PM and can be dispersed into the environment through them. The PM size is decisive in this interaction through the sorption processes (Dos Santos Souza et al., 2021). Although the WHO and EPA guidelines for monitoring air quality are based on the mass concentration of PM, mass is not an ideal metric for risk assessments; it is only a rough indicator of risk. The risk of PM will depend on its chemical composition (Li et al., 2020b) (Table 1). The composition of the particulate material corresponds to a complex mixture of organic and inorganic compounds. Insoluble fraction is in the solid phase and is mainly composed of carbon and ashes derived from lubricants, fuel additives, and engine wear. The soluble fraction, also called organic fraction soluble (OFS), involves organic compounds of high molecular weight and complex in their structure derived from fuels and lubricants. Among them are uncombusted hydrocarbons (alkaline, aromatic), oxygenated hydrocarbons (esters, organic acids, ethers), and polycyclic aromatic hydrocarbons (PAH). The elemental composition of the particulate matter includes different elements, light ones such as aluminum, silicon, potassium, calcium, and heavy ones such as iron, zinc, vanadium, titanium, cadmium, lead, mercury, and antimony (Wang et al., 2012; Raja et al., 2017; Dos Santos Souza et al., 2021).

PM monitors provide a wealth of real-time PM mass concentration data. However, they give no information on the chemical components of the PM. The chemical composition analysis will be carried out from the filter samples. According to the standard, gravimetric analysis should be considered (National Standards Authority of Ireland, 2014). Discard the filter samples that do not show differences in the gravimetric analysis. Proper handling, transportation, and storage of filters are essential to avoid contamination of the filters. The filters should be stored at 4 °C and in the dark until analysis to prevent the loss of semi-volatile organic species and PAHs (Li et al., 2020b; Dos Santos Souza et al., 2021).

For the quantitative analysis of organic and inorganic contaminants, certified reference standards for external calibration are necessary. Using an internal standard is also essential to perform an internal correction of the analysis (Cáceres et al., 2019). Determining the blank values in unexposed filters is necessary to set up the background level of pollution. Glass fibers typically have a relatively high level of contamination, making quantifying the deposited PM composition overestimated (Raja et al., 2017). Ultrasound-assisted extraction combined with solvents is currently a growing tendency for sample preparation in steps of extraction, dissolution, and partial digestion of PM filters. Subsequently, assisted digestion should be used. Ultrasound-assisted extraction plus microwave-assisted digestion provides high digestion of solids, and the microwave system used in this process offers an inert medium for the samples, preventing impurities from leaching onto the container walls (Dos Santos Souza et al., 2021).

For the quantitative analysis of inorganic elements, the most used techniques are inductively coupled plasma mass spectrometry (ICP-MS), atomic absorption spectroscopy (ASS), inductively coupled plasma with atomic emission spectroscopy (ICP/AES), instrumental neutron activation analysis (INAA), photon-induced X-ray fluorescence (XRF), particle-induced X-ray emission (PIXE), and scanning electron microscopy with X-ray fluorescence (SEM/XRF). XRF and PIXE quantify the concentration of elements ranging from sodium (atomic number 11) to uranium (atomic number 92). They are non-destructive techniques; the filter remains intact after analysis and can be used for subsequent analysis by other methods (World Health Organization, 2002).

Water soluble cations (sodium, Na^+^; potassium, K^+^, ammonium, NH^+^) and anions (bromide, Br^−^; fluoride, F^−^; chloride, Cl^−^, nitrite, NO_2_^−,^ nitrate NO_3_^−^, sulphite, SO_3_^−^; sulphate, SO_4_^−^; phosphate, ${\mathrm{PO}}_{4}^{-3}$ ], can be quantified by ion chromatographic and automated colorimetric analysis. AAS and ICP/AES are also appropriate for ion measurements when the PM filter samples are extracted in deionized-distilled water (World Health Organization, 2002; Raja et al., 2017). For the characterization of organic compounds, the gas or liquid chromatography (GC and LC) combined with a mass spectrometer (MS) as a detector can be used. Also, high-resolution mass spectrometry (HRMS; time-of-flight MS [TOFMS], hybrid quadrupole-time-of-flight [QTOFMS], and Orbitrap), often combined with tandem mass spectrometry (MSn), can be used for structural identification. Ultra-high resolution mass spectrometry (UHR-MS) can analyze higher molecular weight organic compounds. High-performance liquid chromatography plus Fourier transform ion-cyclotron-resonance mass spectrometry (HPLC-FTICR-MS) can provide this information if identifying exact elemental compositions is required (Parshintsev and Hyötyläinen, 2015).

Finally, an analysis of the association of all the elements that make up the PM is necessary to understand the health risk associated with exposure to PM. One of the statistical methods used is the multivariate analysis of correlation coefficients. Also, mapping that integrates the direction and speed of the wind provides information on the sources of air pollution by particulate matter. Other meteorological parameters, such as rainfall, humidity, and temperature, can also help assess the variability of particulate matter (World Health Organization, 2002; Cáceres et al., 2019). In addition, mapping that integrates wind direction and speed and other meteorological parameters, such as rainfall, humidity, and temperature, can also help assess particle variability and provide information on sources of air pollution from particulate matter. Having comprehensive information on the concentration and composition of PM, the sources of contamination, and its environmental behavior will help avoid exposure and minimize health risks from PM.

## 6 Conclusion and perspectives

Despite the interesting results that have been reported over the last few years regarding the detrimental effect of PM_2.5_ exposure in the gestational period, the mechanisms that explain how air pollution affects mothers and their offspring, in particular the mechanisms involving the placenta, are largely unknown (Brunst et al., 2022). In addition, there is a scarcity of research articles based on next-generation sequencing and multi-omics technologies on this topic. The application of sequencing-based approaches, such as DNA methylation profiling and histone modification analysis, holds great promise in unraveling the epigenetic alterations induced by PM_2.5_ exposure (Bhatnagar, 2022; Brunst et al., 2022; Zheng et al., 2022; American Heart Association, 2023). The integration of omics sciences and sequencing technologies such as RNAseq, DNAseq, ChIPseq, HiC and Mass Spectrometry in the field of Epigenetics represents a relatively novel and rapidly evolving area of research. Further efforts are needed to expand the utilization of these powerful tools to comprehensively assess the epigenetic changes associated with PM_2.5_ exposure during critical periods of human development. By bridging this knowledge gap, we can gain deeper insights into the molecular mechanisms underlying PM_2.5_-induced epigenetic modifications, enabling the development of targeted interventions and policy measures to mitigate the adverse health effects of air pollution. Due to the highly complex conformation of all PM subtypes, PM_1_, PM_2.5_ and PM_10_, there remain still several open questions, such as 1) how are they internalized in different human tissues and cells? 2) how are their organic and inorganic components further metabolized and transported inside cells, 3) what is the global impact on tissue-specific human epigenome by organic *versus* inorganic PM components? 4) what is the 3D genomic impact of air pollution in human cells? among others. Relative scarce studies have addressed these mechanistic questions with the support from Next-Generation approaches, in due part to the emerging field of Human Exposomics worldwide, as well as the implementation of epigenomic mapping consortia and other multi-scale and muti-disciplinary initiatives, most of which are in consolidation phases. By conducting systematic studies in different cities with different geographic and atmospheric conditions, as multi-country efforts to support vulnerable communities, we should be able to determine early, relative reversible, epigenetic determinants and biomarkers of PM impact in humans for novel therapeutic strategies.
